# ATP-Dependent Chromatin Remodeler CSB Couples DNA Repair Pathways to Transcription with Implications for Cockayne Syndrome and Cancer Therapy

**DOI:** 10.3390/cells14040239

**Published:** 2025-02-07

**Authors:** Rabeya Bilkis, Robert J. Lake, Hua-Ying Fan

**Affiliations:** 1 Biomedical Sciences Graduate Program, University of New Mexico Health Science Center, Albuquerque, NM 87131, USA; rbilkis@fredhutch.org; 2 Program in Cell and Molecular Oncology, University of New Mexico Comprehensive Cancer Center, Albuquerque, NM 87131, USA; rjlake@salud.unm.edu; 3 Division of Molecular Medicine, Department of Internal Medicine, University of New Mexico Health Science Center, Albuquerque, NM 87131, USA

**Keywords:** CSB, PARP1, PARP2, Cockayne syndrome, cancer therapy, TC-NER, TA-SSBR, transcription, DNA repair

## Abstract

Efficient DNA lesion repair is crucial for cell survival, especially within actively transcribed DNA regions that contain essential genetic information. Additionally, DNA breaks in regions of active transcription are prone to generating insertions and deletions, which are hallmark features of cancer genomes. Cockayne syndrome protein B (CSB) is the sole ATP-dependent chromatin remodeler that is essential for coupling DNA repair pathways with transcription, leading to more efficient DNA repair in regions of active transcription. CSB is best known for its essential function in transcription-coupled nucleotide excision repair (TC-NER), a process that rapidly removes helix-distorting DNA lesions that stall RNA polymerase II, such as those created by chemotherapeutic platinum compounds and UV irradiation. In addition to NER, CSB has also been reported to couple homologous recombination to transcription. Most recently, CSB has also been shown to couple single-strand DNA break repair to transcription. In this review, we will discuss the overlapping and distinct mechanisms by which CSB couples these different DNA repair pathways to transcription. We will also discuss how these CSB functions may account for Cockayne syndrome and the emerging roles of CSB as an innovative target for cancer therapy.

## 1. Introduction

**Figure 1:**
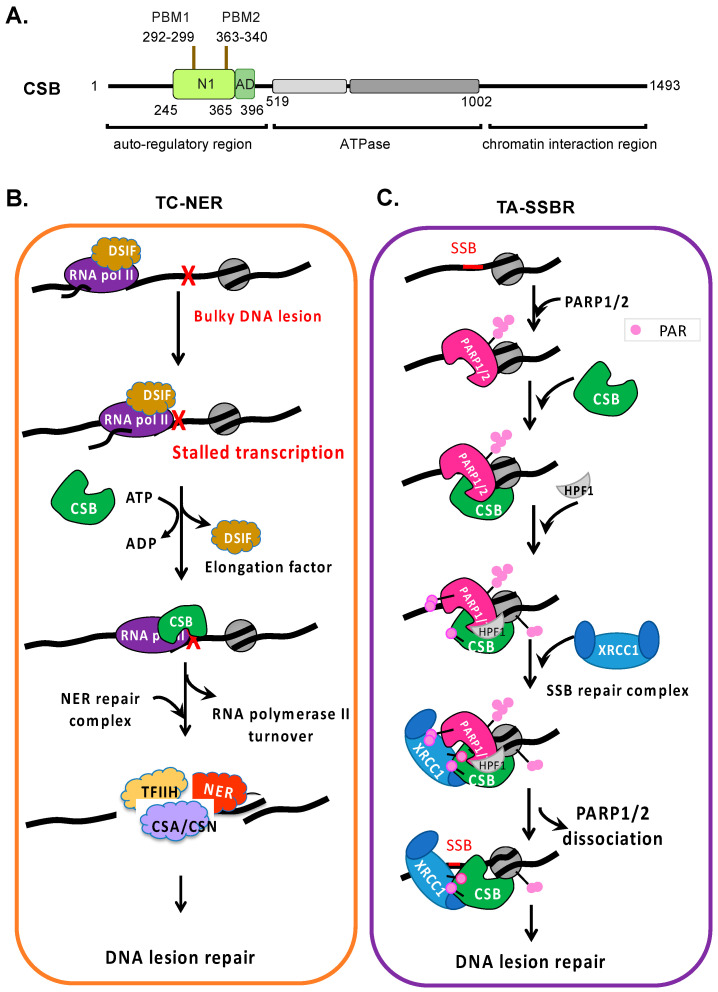
Coupling of NER and SSBR to transcription by CSB. (**A**) Schematic of CSB’s functional domains. PBM: PARylation binding module. AD: acidic domain. N1: coupling chromatin remodeling to ATPase activity. (**B**) A minimalized TC-NER model. CSB locates bulky DNA lesion-stalled transcription in an ATP-dependent manner. ATP hydrolysis by CSB is predicted to induce a conformational change that permits stable chromatin association. CSB replaces DSIF, transitioning polymerase II from a transcription elongation complex to a DNA repair complex. CSB is essential for the rapid recruitment of the NER protein as compared to GG-NER. ATP-dependent chromatin remodeling by CSB may enhance the DNA repair efficiency by altering the chromatin environment and stimulating the recruitment of NER proteins. (**C**) A proposed TA-SSBR model. Single-stranded DNA breaks are detected and bound by PARP1 and PARP2, which activates their enzymatic activity. CSB is then recruited through direct interaction with PARP1 or PARP2, promoting the recruitment of HPF1, which alters the substrate specificity of PARP1 and PARP2, most notably facilitating histone PARylation, which likely promotes chromatin relaxation to facilitate DNA repair. XRCC1-containing SSBR complexes are then rapidly recruited by CSB and PARylated PARP1, PARP2, and histones. CSB may likely facilitate the dissociation of PARP1/2 from DNA breaks to promote the rapid progression of DNA repair in transcribed DNA regions.

Genome integrity maintenance is crucial for healthy aging and disease prevention [1]. It is particularly important at actively transcribed genomic regions, where genome integrity loss can impact protein function, leading to cell death or disease [2,3,4,5]. Cockayne syndrome protein B (CSB) is a member of the Snf2/Swi2 ATPase-dependent chromatin remodeler family (Figure 1A) [6]. Indeed, Nicolai and collaborators have demonstrated that the CSB protein is involved in maintaining the dynamics and integrity of chromatin [7]. ATP-dependent chromatin remodelers are modular in structure, with the central region containing an ATPase domain consisting of seven conserved helicase motifs. The regions flanking the ATPase domain are critical for targeting and activity regulation [8]. They exhibit DNA- and/or nucleosome-stimulated ATP hydrolysis activity but no detectable helicase activity (i.e., DNA strand separation). These proteins use ATP as energy to alter DNA–histone contacts and regulate the chromatin structure to regulate fundamental nuclear processes, such as DNA repair and transcription [9]. Some family members can also alter the DNA contacts of non-histone proteins, such as transcription factors [9].

Cockayne syndrome is a progressive premature aging disease associated with developmental and neurological disorders [10,11,12,13]. Mutations in the *ERCC6* gene that encodes the CSB protein account for the majority of Cockayne syndrome cases. Other mutations that lead to Cockayne syndrome occur in the *ERCC8* gene that encodes the CSA protein and, most rarely, genes that encode components of the TFIIH transcription factor complex. CSB is involved in multiple cellular processes—most notably, DNA repair, transcription, mitochondrial metabolism, and the hypoxic response [6,14,15,16,17,18,19,20,21,22,23]. Importantly, CSB is the only ATP-dependent chromatin remodeler demonstrated to directly couple DNA repair to transcription.

## 2. CSB Couples DNA Repair to Transcription

CSB is best known for its essential role in transcription-coupled nucleotide excision repair (TC-NER), in which CSB locates stalled transcription generated by bulky DNA lesions that distort the DNA helix and recruits canonical TC-NER repair proteins (Figure 1B) [24]. CSB has also been suggested to participate in the repair of erroneously modified DNA bases, as well as in coupling the homologous recombination repair of DNA double-strand breaks to transcription [25]. Most recently, CSB has been shown to play a critical role in poly(ADP-ribose) polymerase 1 and 2 (PARP1 and PARP2)-mediated single-strand break repair at actively transcribed DNA regions (Figure 1C) [26]. Here, we compare and contrast the mechanisms by which CSB couples DNA repair to transcription, the implications of these transcription–repair coupling mechanisms for Cockayne syndrome, and how these mechanisms might be exploited for cancer therapy.

### 2.1. CSB’s Roles in Transcription-Coupled Nucleotide Excision Repair

TC-NER is a subpathway of nucleotide excision repair that preferentially repairs bulky helix-distorting DNA lesions that stall transcription, such as UV-generated cyclobutane pyrimidine dimers (CPD), (6-4) pyrimidine-pyrimidone photoproducts (6-4PP), and crosslinking chemotherapeutic drugs such as cisplatin, carboplatin, oxaliplatin, and nitrogen mustard [24,27,28]. More recently, aldehyde-induced DNA–protein crosslinks have also been shown to be CSB-mediated repair targets (Figure 1B) [29,30]. TC-NER repairs DNA lesions much faster than global genome NER (GG-NER), which is CSB-independent but xeroderma pigmentosum complementation group C (XPC)-dependent. Without CSB, there is no preferential repair of the transcribed DNA strand, and GG-NER is used as the default NER mechanism [3]. Venema and colleagues first showed that, upon UV-induced DNA damage, fibroblasts derived from Cockayne syndrome patients had slower repair kinetics on the transcribed DNA strand as compared to normal fibroblasts, which had faster repair kinetics on the transcribed DNA strand than the non-transcribed DNA strand [31]. Troelstra and colleagues subsequently cloned the excision repair cross-complementation *ERCC6* gene that encodes the CSB protein, based on its ability to complement the repair defect of an *ERCC6* mutant cell line [32,33]. Although first characterized in mammalian cells, the importance of the preferential repair of actively transcribed DNA is underscored by the evolutionary conservation of TC-NER across organisms, ranging from bacteria and yeast to humans.

#### 2.1.1. ATP Hydrolysis by CSB Is Essential to Locate DNA Lesion-Stalled Transcription

The CSB protein interacts with chromatin dynamically but becomes stably associated with chromatin in UV-treated cells [34,35]. CSB mutants defective in ATP binding/hydrolysis fail to locate lesion-stalled transcription and, thus, do not stably associate with chromatin upon UV-induced DNA damage, revealing that targeting during TC-NER is an ATP-dependent process. Interestingly, a CSB derivative without its N-terminal 454 amino acids loses its substrate specificity and binds to chromatin constitutively, demonstrating that the N-terminal region is critical for substrate specificity (Figure 1A) [35]. The current model is that ATP hydrolysis by CSB exposes a substrate recognition domain in its N-terminal region, the essential first step of TC-NER (Figure 1B). CSB binding to lesion-stalled transcription is then stabilized through chromatin contacts involving its ATPase domain and C-terminal region. In the absence of bulky DNA adducts and stalled RNA polymerase II, the N-terminal region functions as an auto-inhibitory domain, preventing productive chromatin associations. Excitingly, recent structure studies have revealed that CSB and the transcription regulator DRB sensitivity-inducing factor (DSIF) bind to overlapping sites on RNA polymerase II [36,37]. The replacement of DSIF with CSB appears to be a critical step in the conversion of RNA polymerase II from a transcription elongation complex to a DNA repair complex (Figure 1B) [38,39].

#### 2.1.2. Recruitment of NER Factors Is Initiated by CSB Through Protein–Protein Interactions

Upon locating a DNA lesion-stalled RNA polymerase II, CSB recruits the Cockayne syndrome group A protein (CSA), a component of an E3 ubiquitin ligase complex that contributes to RNA polymerase II turnover [40,41] and the recruitment of nucleotide excision repair factors [37,42]. Using a separation-of-function CSB derivative, CSB∆N1 (CSB∆245–365, Figure 1A), which is active for ATP hydrolysis but unable to reposition nucleosomes, Cho and colleagues revealed that CSB∆N1 is able to locate DNA lesion-stalled transcription and recruit key NER factors. Importantly, this study revealed that CSB recruits NER proteins through direct protein–protein interaction and is independent of nucleosome repositioning by CSB [37]. Given that CSB∆N1 does not fully complement the UV sensitivity of CSB-null cells, these observations indicate that chromatin remodeling by CSB has additional functions during TC-NER, such as generating a chromatin environment that supports efficient repair or promoting transcription resumption after repair.

### 2.2. CSB Regulates Oxidative DNA Lesion Repair

Oxidative stress underlies numerous pathologies, including cancer, aging, inflammation, and neurodegenerative diseases [43,44]. Reactive oxygen species (ROS) are constantly generated during normal cellular metabolism [45]. The hydroxyl radical is the major cause of oxidative DNA damage; it attacks both the DNA bases and the sugars of the phosphodiester backbone. These DNA lesions are repaired by base excision repair (BER) and single-strand break repair (SSBR), respectively [46,47,48]. These pathways differ only in the initial recognition and processing of the DNA lesion, with shared subsequent steps [49,50].

#### 2.2.1. CSB Facilitates Base Excision Repair

The BER pathway repairs DNA base damage, such as base alkylation, oxidation, and deamination [51]. It is initiated when an enzyme known as a DNA glycosylase recognizes the damaged base. The recognition of a damaged base such as 8-oxoguanine, a highly mutagenic derivative, by the 8-oxoguanine DNA glycosylase (OGG1), a major BER enzyme, causes the cleavage of the N-glycosidic bond between the base and sugar phosphate backbone, removing the damaged base and creating an apurinic/apyrimidinic (AP) site (i.e., abasic site) [52,53]. OGG1 also has lyase activity that can cleave the DNA backbone, necessary to initiate repair, albeit with slow kinetics [54]. Dedicated lyases can also assist OGG1 in incising the DNA backbone. Some other glycosylases, which do not have lyase activity and only create an apurinic/apyrimidinic (AP) site, need to work in concert with an AP endonuclease, such as APE1 and APE2. In short-patch BER, the single-strand break is converted to a gap by the lyase activity of DNA polymerase β, which then adds a base through its polymerase activity. After base addition, the single-strand break is sealed by DNA ligase III. Another BER pathway, long-patch repair, utilizes DNA polymerase δ and polymerase ε, as well as the PCNA processivity factor, to extend the repair patch and displace the damaged single-strand DNA fragment. The displaced segment is then removed by a flap endonuclease, generating a single-strand break that is sealed by DNA ligase I.

CSB’s importance in oxidative DNA damage repair was revealed in cells that lacked functional CSB, which displayed increased sensitivity to oxidizing agents such as hydrogen peroxide (H_2_O_2_), menadione, and γ-irradiation-induced oxidative stress [22,26,55,56,57]. Like UV irradiation, oxidative stress stabilizes CSB’s interaction with chromatin [22,58]. Several studies suggest that CSB facilitates BER [59]. Dianov and colleagues observed that cells with mutant CSB had an ineffective OGG1 incision, and this inefficiency was reversed when the cells were reconstituted with the wild-type *CSB* gene [60]. Additionally, studies showed that CSB-null mice accumulated more 8-oxoguanine lesions than mice with functional CSB [56,61]. CSB and OGG1 are found in the same protein complex, although no direct CSB–OGG1 interaction has been reported [62,63]. Moreover, CSB physically interacts with APE1, a base excision repair enzyme, and stimulates APE1 activity in an ATP-independent manner [64]. In contrast, while oxidative stress induces a stable CSB–chromatin association, reducing the OGG1 and APE1 levels using shRNA does not alter the kinetics of the CSB–chromatin association in response to oxidative stress. These observations suggest that CSB is not directly coupled to OGG1 and APE1 function in BER [22]. Furthermore, Menoni and colleagues showed that CSB facilitates the recruitment of the scaffold protein X-ray repair cross-complementing protein 1 (XRCC1), which is essential for SSBR, to laser-assisted locally generated 8-oxoG lesions, promoting DNA base damage repair [65]. These results together indicate that CSB functions in SSBR upon oxidative DNA damage and may facilitate BER at a downstream level, focusing on SSBR.

#### 2.2.2. CSB Facilitates Single-Strand DNA Break Repair

**Figure 2:**
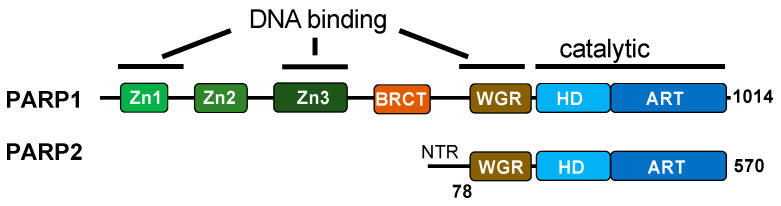
Functional domain comparison between PARP1 and PARP2. PARP1 binds to SSBs with a variety of end chemistries through its zinc fingers and Trp-Gly-Arg (WGR) domain. The zinc fingers are absent in PARP2, which binds primarily to the 5′ phosphate ends of SSBs through its WGR domain and N-terminal region (NTR). The WGR domain, present in both PARP1 and PARP2, is critical in inducing conformational changes and the allosteric regulation of enzymatic activity.

Unlike the BER pathway, which initiates with DNA glycosylases, the repair of direct SSBs begins with DNA break recognition by PARP1 and PARP2 (Figure 1C and Figure 2) [66,67]. PARP1 recognizes SSBs using zinc fingers and the WGR domain, binding to conventional and unconventional DNA ends [68,69]. On the other hand, PARP2 binds SSBs through the WGR domain alone, with a preference for 5′-phosphorylated DNA ends [70]. SSB binding induces a conformational change in PARP1, exposing its NAD^+^ binding site, which leads to the allosteric activation of its enzymatic activity [69]. In contrast, PARP2 activation may require additional factors beyond DNA binding, as suggested in a recent study [71].

After binding to the DNA break, PARP1 and PARP2 transfer ADP-ribose from nicotinamide adenine dinucleotide (NAD+) to themselves and neighboring proteins to form poly(ADP-ribose) (PAR) polymer chains. The PAR polymer acts as a beacon to signal the recruitment of repair proteins and their assembly at the damaged site. The scaffold protein XRCC1 is recruited to PARylated proteins—most notably auto-PARylated PARP1 [72]. XRCC1 brings with it associated repair enzymes, such as polynucleotide kinase-phosphatase, DNA polymerase β, and DNA ligase III. More recently, histone PARylation factor 1 (HPF1) was found to be a regulator of PARP1 and PARP2 catalytic activity [73,74,75,76,77]. HPF1 alters the PARP1 and PARP2 substrate specificity from primarily aspartate and glutamate to serine residues during DNA damage and facilitates histone PARylation. Histone PARylation is known to relax the chromatin structure [78], which may play an important role in repair protein accessibility. Importantly, PARP1 and PARP2 must dissociate from SSBs to allow DNA repair to ensue [79]. PARP1 and PARP2 autoPARylation is critical for the dissociation of PARP1 and PARP2 from chromatin, mediated by electrostatic repulsion as the PAR chains and DNA are both negatively charged [68].

##### CSB Interacts with PARP1 at Sites of DNA Breaks

Thorslund and colleagues showed that CSB directly interacts with non-PARylated and PARylated PARP1 in vitro and in cells [80]. Moreover, oxidative stress induces the colocalization of CSB with PARP1 and PAR, as shown by immunostaining approaches, suggesting a functional interaction between these two proteins during oxidative stress. Using protein–chromatin co-fractionation, Boetefuer and colleagues showed that PARP1 facilitates the association of CSB with oxidized chromatin, although the PARylation activity of PARP1 was not essential for this association, consistent with previous observations [22]. Together, these results indicate that CSB collaborates with PARP1 in SSBR.

Using chromatin immunoprecipitation followed by deep sequencing (ChIP-seq), CSB was found to be highly enriched at four genomic loci in oxidatively stressed cells [58]. Strikingly, PARP1 was found to be essential in recruiting CSB to these loci, suggesting that these regions are hotspots for DNA lesions generated during oxidative stress and can function as surrogate loci for the study of PARP1–CSB function [55]. Indeed, using anti-PARP1 ChIP-qPCR to locate PARP1 at these regions, and ADP-ribose chromatin affinity precipitation (ADPr-ChAP) to detect PARP1 activity, PARP1 was found present at these regions in a CSB-dependent manner upon the onset of oxidative stress (~20 min). PARP1 subsequently dissociated from these regions (~40 min). Interestingly, the PAR chains and the CSB protein remained for a longer period than PARP1. These results are consistent with a model in which PARP1 recruits CSB to DNA lesions, CSB stabilizes PARP1 at DNA lesions, and they together recruit the SSBR protein machinery. Ultimately, CSB facilitates PARP1’s dissociation from oxidized chromatin to allow SSBR to ensue (Figure 1C) [55].

##### CSB Facilitates SSBR Mediated by PARP1 and PARP2, Predominantly at Genomic Regions with Active Transcription

Using alkaline comet assays to monitor DNA break repair, Bilkis and colleagues demonstrated that CSB facilitates oxidative DNA repair in an ATP-dependent manner [26]. Strikingly, transcription inhibition with α-amanitin (which stalls RNA polymerase) or 5,6-dichloro-1-beta-D-ribofuranosylbenzimidazole (DRB) (which clears the coding template of elongating RNA polymerase II) bypassed CSB’s function in oxidative DNA repair, indicating that CSB primarily repairs single-strand DNA breaks in regions of active transcription. This study also showed that PARP1 repairs oxidative DNA lesions regardless of the local transcription status, but PARP2, like CSB, predominantly functions in SSBR at actively transcribed DNA regions. These results were supported by co-immunoprecipitation experiments where oxidative stress was shown to induce CSB interactions with PARP1 and PARP2. Once recruited to oxidized chromatin, CSB was shown to facilitate the recruitment of downstream repair proteins, such as HPF1 and XRCC1 [26,55,65]. Thus, this study uncovered a novel subpathway of SSBR that preferentially repairs SSBs at actively transcribed DNA and is regulated by CSB (Figure 1C) [26].

### 2.3. CSB Couples Homologous Recombination Repair to Transcription in Oxidatively Stressed Cells

CSB has also been shown to facilitate the repair of DSBs generated during transcription in oxidatively stressed cells [25,81,82]. During active transcription, RNA:DNA hybrids form between the nascent transcript and the coding DNA strand, with the noncoding DNA strand displaced. If these structures are not resolved into duplex DNA with displaced RNA, they form a structure termed an R loop [83]. R loops are promoted by the formation of G-quadruplex (G4) structures on the displaced, noncoding strand [82]. Both R loops and G4 structures lead to increased genome instability [84]. CSB has been suggested to sense R loops through the recognition of methylated cytosine residues (methyl-5-cytosine, or m^5^C) on the nascent RNA transcript, promoting processing by a mechanism termed transcription-coupled homologous recombination (TC-HR). The m^5^C modification of the RNA inhibits the initiation of PARP1-mediated non-homologous end joining (NHEJ), an error-prone double-strand DNA repair mechanism, ensuring that error-free HR will repair the lesion. Given that HR normally requires sister chromatids, TC-HR would be largely restricted to the S and G2 phases of the cell cycle. TC-HR, however, differs from traditional HR as it appears to be BRCA-independent (for a more detailed review, see [85]). The model proposed by Keskin and colleagues has the nascent RNA template annealing to the damaged ends at DSBs in a Rad52-dependent manner, which promotes strand ligation in a reverse transcriptase-dependent manner [81]. The TC-HR model would allow DSB repair to occur in the G1 phase or in terminally differentiated cells [81,82]. Additionally, the NER protein XPF can also be recruited by RAD52 to resolve the G4 structure present at R loops, aiding in R loop disassembly through decreased G4 stabilization. Overall, TC-HR requires the recognition of R loop structures at sites of DSB stalled transcription by CSB. This, in turn, inhibits PARP1 activation, prevents NHEJ repair, and, through the regulation of HR protein recruitment, initiates BRCA-independent HR.

## 3. Interaction of CSB and CTCF, the Master Regulator of the Three-Dimensional Chromatin Structure

During DNA repair, the three-dimensional chromatin structure is rearranged not only around the damaged regions but across the genome to allow for increased interactions between the damaged DNA and repair factors [86,87]. The CCCTC-binding factor (CTCF) plays an indispensable role in regulating long-range interactions by promoting the formation of DNA loops [88,89]. Studies using chromosome conformation capture sequencing (Hi-C or 3C sequencing) have revealed that topologically associating domains (TADs) are altered upon DNA daamage, suggesting a role for CTCF in the DNA repair response [86,87,90,91,92,93].

In an effort to understand the regulation of SSBR in the context of the higher-order chromatin structure, Lake and colleagues demonstrated that oxidative stress induces CSB occupancy at genomic regions containing the CTCF-binding motif [58]. Surprisingly, CSB facilitated CTCF occupancy at CSB-occupied sites that lacked a CTCF-binding consensus, suggesting the coordination of functional regulation between CSB and CTCF. Additionally, it was found that oxidative stress promoted an interaction between the CSB and CTCF proteins. These observations support a model in which CSB collaborates with CTCF to reorganize the chromatin structure through their respective activities, thereby promoting efficient oxidative DNA damage repair.

Recent evidence suggests that the repair of DNA breaks occurs in biomolecular condensates [94]. Condensates are subcellular compartments without physical membrane boundaries that compartmentalize biochemical reactions by concentrating various molecules, including proteins and nucleic acids [92,93,94,95]. Indeed, condensates increase the local concentrations of DNA damage response molecules within specific nuclear foci to possibly coordinate the dynamics of the DNA repair processes [94,95]. PARP1 forms biomolecular condensates through phase separation while bound to DNA, a process dependent on its zinc finger domains [96]. PARylation increases PARP1 condensation in a chain-length-dependent manner, influencing the internal dynamics of these condensates. Importantly, DNA repair proteins partition differently within PARP1 condensates, and this partitioning facilitates DNA repair [96]. CTCF also forms condensates or regulatory hubs that organize the three-dimensional structure of chromatin [97]. Given the association between CSB, CTCF, and PARP1, CSB may collaborate with both CTCF and PARP1 to form DNA repair hubs to promote the efficient repair of SSBs in oxidatively stressed cells.

## 4. CSB Function in Neuron Development and Neurodegeneration

Defects in SSBR are directly linked to abnormalities in brain function [49,98,99,100]. In adult mammals, neurons remain in a post-mitotic, terminally differentiated state. Studies show that, while non-dividing neurons can repair transcribed genes, they nonetheless exhibit reduced global DNA repair, and the accumulation of damage in transcribed regions can lead to a variety of neuropathies [101]. Additionally, the accumulation of DNA damage with age can cause altered gene expression, resulting in neuronal dysfunction. Indeed, neuronal activity alone can lead to the production of DNA breaks [99,102]. Using a targeted sequencing technique that identified DNA repair sites (Repair-seq), Reid and colleagues showed that open chromatin, including gene bodies and active regulatory regions, served as hotspots for DNA repair in human embryonic stem cell-induced neurons [99]. This sequencing technique leverages the common requirement for DNA synthesis to complete most DNA repair pathways. A similar technique, synthesis associated with repair sequencing (SAR-seq), modified for single-strand break detection, revealed that post-mitotic neurons spontaneously accumulate a large number of single-strand breaks, which are localized to enhancers in proximity to CpG dinucleotides and demethylation sites [100]. Interestingly, this study revealed that PARP1 and XRCC1 are instrumental in the repair of these SSBs. Several neurodegenerative diseases are associated with the defective repair of single-strand breaks and double-strand breaks [21,49,103,104], and patients with Cockayne syndrome exhibit various neurological disorders, including cerebellar dysfunction, sensorineural hearing loss, microcephaly, cognitive retardation, and optic atrophy [3,9,97]. Indeed, studies indicate the dysregulation of neuron-specific gene expression in CSB-deficient cells as the cause of Cockayne syndrome [14,105,106,107]. We suggest that CSB-mediated TA-SSBR is also critical for neuronal health, and impaired TA-SSBR contributes significantly to the neurological disorders associated with Cockayne syndrome patients.

## 5. CSB Is a Cancer Prognostic Marker and an Emerging Target for Cancer Therapy

CSB has been suggested as a target for cancer therapy [18,20,108,109,110,111]. In vitro viability assays have demonstrated that reducing the CSB protein levels sensitizes cancer cells to platinum-based chemotherapeutic agents and PARP inhibitors [80,108]. Studies from the Molecular Taxonomy of Breast Cancer International Consortium (METABRIC) indicate that lower CSB levels correlate with better overall survival in breast cancer patients [112]. This is likely due to CSB’s role in coupling transcription to DNA repair, countering the DNA damage effects of therapeutic agents. Importantly, these observations highlight CSB’s potential as an innovative therapeutic target in cancer treatment.

PARP inhibitors have been effective in treating homologous recombination (HR)-deficient breast and ovarian cancers based on the synthetic lethality observed between PARP inhibitors and cancer cells deficient in HR [113,114,115,116,117]. Two major mechanisms have been proposed to account for this synthetic lethality. First, PARP inhibitors increase the residence time of PARP1 on DNA, which can lead to inefficient DNA repair, and current data suggest that CSB can promote PARP1/2 dissociation from damaged chromatin. Second, the enzymatic activity of PARP1 has been shown to be important in resolving DNA damage created by transcription–replication conflicts (TRC) [118]. It will be of great interest to determine whether CSB also collaborates with PARP1 in resolving TRCs.

As discussed above, DNA breaks activate PARP1 and PARP2, leading to their auto-PARylation, which facilitates their dissociation from DNA lesions, allowing repair to proceed [79]. Biochemical studies indicate that HPF1 increases PAR chain branching and decreases the PAR chain length, which increases the residence time of PARP proteins on damaged chromatin. These observations suggest that additional mechanisms are needed to facilitate the dissociation of PARP1 and PARP2 from damaged chromatin [71]. For example, the ATP-dependent chromatin remodeler Amplified in Liver Cancer-1 (ALC1) contains a macrodomain that binds mono-ADP-ribosylated proteins and has been found to modulate PARP2–chromatin retention, thereby facilitating DNA repair [119]. Indeed, the binding of the ALC1 macrodomain to PAR activates its chromatin remodeling activity and, importantly, ALC1 deficiency increases the sensitivity to PARP inhibitors [120].

CSB is also a PAR reader, containing two PAR-binding motifs (PBMs) in its N1 region (Figure 1A) [121,122]. Cho and colleagues have shown that the remodeling activity of CSB is tunable, as NAP1-like histone chaperones bind to the CSB N1 region and enhance its chromatin remodeling activity 10-fold [37]. It would be of great interest to determine whether PAR enhances CSB remodeling activity and whether CSB can facilitate the dissociation of PARP1 or PARP2 from oxidized chromatin after SSBR machinery recruitment, allowing DNA repair to ensue efficiently. Indeed, using PARP1 ChIP-qPCR, Lake and colleagues observed that CSB stabilizes the chromatin association of PARP1 upon the onset of single-strand break signaling yet also facilitates its subsequent dissociation from chromatin, a prerequisite for SSBR to proceed (Figure 1C) [55]. This observation suggests parallels between ALC1 and CSB functions in PARP-associated DNA repair.

Developing PARP inhibitors that are more specific to PARP1 is critical in reducing the toxicity to improve cancer therapy [123]. One type of toxicity associated with current PARP inhibitors arises from the lack of specificity between PARP1 and PARP2. PARP2 plays an essential role in erythropoiesis, which has been suggested to account for some of the toxicity of PARP inhibitors [124,125]. As mentioned earlier, SSB binding induces a conformational change in PARP1, exposing its NAD+ binding sites, which leads to the allosteric activation of its enzymatic activity [69]. In contrast, PARP2 activation may require additional factors beyond DNA binding [71]. Given that CSB and PARP2 have been observed to play an essential role in TA-SSBR [26], it is plausible to propose that CSB may enhance PARP2 activation at DNA breaks. Accordingly, elucidating the distinct regulatory roles of CSB in SSBR-mediated by PARP1 and PARP2 might uncover novel cancer therapeutic strategies that maximize PARPis’ efficacy with reduced toxicity to improve patient outcomes in cancer treatment.

In conclusion, CSB plays an essential role in promoting the efficient repair of a variety of DNA lesions in actively transcribed DNA regions. This presents a unique opportunity to target CSB for cancer treatment in combination with platinum-based and PARP inhibitor therapies.

## Data Availability

No new data were created or analyzed in this study. Data sharing is not applicable to this article.
